# Implementation of evidence-based guidance for dementia palliative care using participatory action research: examining implementation through the Consolidated Framework for Implementation Research (CFIR)

**DOI:** 10.1186/s43058-021-00241-7

**Published:** 2021-12-11

**Authors:** Alice Coffey, Irene Hartigan, Suzanne Timmons, Catherine Buckley, Elaine Lehane, Christina O’Loughlin, Selena O’Connell, Nicola Cornally

**Affiliations:** 1 Health Implementation Science and Technology Cluster, Health Research Institute, University of Limerick, Limerick, Ireland; 2 Department of Nursing and Midwifery, University of Limerick, Limerick, Ireland; 3 Catherine McAuley School of Nursing and Midwifery, University College Cork, Cork, Ireland; 4 Centre for Gerontology and Rehabilitation, School of Medicine, University College Cork, Cork, Ireland; 5 Northridge House Education and Research Centre, St Lukes Nursing Home, Cork, Ireland

**Keywords:** Implementation, Conceptual framework, CFIR, Participatory action research, Dementia palliative care, Long-term care

## Abstract

**Background:**

The importance of providing evidence-based palliative care for people with dementia is increasingly acknowledged as important for patient outcomes. In Ireland, evidence-based guidance has been developed in order to address key features of dementia palliative care, including the management of pain, medications and hydration and nutrition. The aim of this study was to identify and explore the factors affecting the implementation of evidence-based guidance on dementia palliative care.

**Methods:**

The Consolidated Framework for Implementation (CFIR) guided a mixed-method pre-post study. One guidance document pertaining to the management of pain, medication or hydration and nutrition was implemented in three long-term care facilities. Participatory action research in the form of work-based learning groups was used to implement the guidance, drawing on a situational analysis (pre-implementation). Staff questionnaires and audits were conducted pre- and post-implementation while champion interviews were also conducted post-implementation.

**Results:**

Features of the guidance, the inner setting components such as readiness to change, and the process of implementation were most frequently identified as impacting implementation. Components of the outer setting, such as external policy incentives and individual characteristics, featured less commonly. Data from qualitative interviews revealed that the guidance was perceived as advantageous or complimentary to previous care provided. Within the inner setting, leadership and support from other colleagues facilitated implementation. However, limited availability of other healthcare professionals to assist with carrying out guidance actions presented a barrier in some facilities. The external facilitators of the work-based learning groups (WBLGs) were perceived as experienced and encouraged active participation and reflection on practices. Despite the challenge of releasing staff to attend the WBLGs, quantitative data demonstrated reduced staff de-motivation amongst those who did attend was noted post-implementation (pre-*Mdn* = 19.50 versus post-*Mdn* = 22.00, *U* = 497.00, *p* = 0.07).

**Conclusions:**

A situational analysis informed by the CFIR framework in conjunction with a participatory action research approach helped to advance the implementation of the guidance. The progress of implementation depended on the extent to which evidence-based care was previously being implemented at each site. Post-implementation analysis using CFIR identified challenges to address in future projects such as staff cover and timing of training to facilitate attendance for staff with different working hours. Facilitators included multidisciplinary engagement with the intervention and champions at each site to support the implementation process.

**Supplementary Information:**

The online version contains supplementary material available at 10.1186/s43058-021-00241-7.

Contributions to the literature

- This study combined the Consolidated Framework for Implementation Research (CFIR) with a participatory action research approach in a tailored implementation strategy to implement evidence-based guidance for dementia palliative care.
- A combined implementation strategy tailored to the care setting context may reduce staff de-motivation towards readiness for change for practice relating to dementia palliative care.
- This paper identifies the influential components associated with the effective implementation of evidence-based guidance for dementia palliative care within long-term care settings.

## Background

Dementia is a degenerative, life-limiting illness that affects approximately 50 million people worldwide [1]. Internationally, there is an emphasis on improving dementia care including dementia palliative care in long-term care [2]. The benefits of palliative care for dementia, in symptom management and improvements in patient outcomes such as quality of life at end of life, have been confirmed [3]; however, dementia palliative care is complex [4]. People with advanced dementia commonly experience pain, eating and swallowing problems, and behavioural symptoms [5–7]. Assessment and management of these symptoms is challenging given the existing cognitive and communication impairment and comorbid conditions [5, 8, 9]. In 2016, the Irish Hospice Foundation launched guidance documents for dementia palliative care, three of which targeted hydration and nutrition [10], pain assessment and management [11] and medication management [12]. Poor management of pain, medications and nutrition can negatively affect residents through adverse drug reactions, reduced quality of life and increased risk of mortality [6–8], underscoring the importance of advancing evidence-based practice in these areas.

However, integrating evidence-based guidance into practice is complex and often insufficiently achieved [13, 14]. A growing area of literature seeks to understand implementation processes in order to advance the implementation of healthcare guidelines [13–15]. However, many factors influence implementation. There is no single theory or set of theories that offer testable hypotheses about the constructs that predict specific outcomes within implementation science [16–18], and organisations have a complex web of relationships [17]. However, there are several frameworks that identify typologies of factors, hypothesised to affect implementation outcomes [17]. The Consolidated Framework for Implementation Research (CFIR) is a determinant-style framework that seeks to identify the barriers and facilitators of implementation [19]. Researchers can draw on domains and sub-domains from the CFIR that are most relevant for their project [19].

Literature on the implementation of evidence-based guidance for dementia care in nursing homes identified factors influencing implementation, such as relevance to the staff’s role and practice; interactive group work, face-to-face discussions and experiential learning; high-quality training materials; and experienced and trainee-centred facilitators [20]. Research by Surr et al. [21] using the Behaviour Change Wheel [22] found that opportunity factors were the most prevalent barriers including lack of time, staff and other resources. However, little is known about the factors influencing the implementation of dementia palliative care specifically.

Participatory action research (PAR) can be used to target factors influencing the implementation of educational interventions in dementia care. PAR involves a process of self-reflective inquiry where participants reflect on and improve their practices [23]. Addressing the factors identified as influential in dementia care education, PAR places emphasis on knowledge creation so that practices are relevant to the context, problem-based, experiential and involving active facilitation [23–25]. While characteristics of PAR are similar to the facilitators of dementia education [20], there is limited research on PAR for dementia palliative care in the long-term care setting or on determinants of implementation in this setting. This project used a PAR approach with researchers and participants collaborating to implement evidence-based guidance on dementia palliative care and evaluate the process of implementation. This research investigates the implementation using the PAR approach and CFIR as a guiding framework.

## Methods

The CFIR framework guided a pre-post, mixed-method design to identify and explore the factors affecting the implementation of evidence-based guidance for dementia palliative care in long-term care settings. The pre-implementation situational analysis informed the PAR approach to implementation [26]. The Standards for Reporting Implementation Studies (StaRI) statement was used to aid reporting of the study [27], and the recommendations of Proctor et al. [28] were used to identify and report the implementation strategies. Ethical approval was obtained from the University Clinical Research Ethics Committee (log number: ECM4 (oo) 5/6/18 & ECM 3(nnnn)3/7/18).

### Dementia palliative care evidence-based guidance

The intervention consisted of implementing one of three evidence-based guidance (hereinafter known as the guidance) for dementia palliative care in each of three long-term care facilities: hydration and nutrition [10], pain assessment and management [11] and medication management [12]. The guidance followed the broad principles of guideline development outlined by the Irish Department of Health [29]: scoping review of literature, collation of key themes, internal group consultation and wide external consultation with key stakeholders, nationally and internationally. The guidance followed a similar format, including fact sheets and links to resources [10–12].

### Setting

Three long-term care residential facilities for older people in the South of Ireland were recruited. The primary inclusion criterion was the provision of residential care predominantly (but not exclusively) to persons with dementia. Three facilities were selected in collaboration with the Area Manager for Older Person Services in the region based on size, capacity to engage in research, location and existing education/research relationship with the research team. Each Director of Nursing was provided with an invitation letter which contained details regarding the study and ethical approval procedures. Once formal expression of interest to partake in the study was received, the research team met with the Directors to discuss the plan of research and level of commitment required from each facility. The intervention was applied on one ward within two larger sites and through a whole setting approach in the remaining site. The number of resident beds on study sites ranged from 17 to 33 and the proportion of residents diagnosed with dementia varied from 42 to 88% in study sites and this information was obtained from the case notes in collaboration with the attending Consultant Geriatrician. Details on facility characteristics are published elsewhere [25]. Care was primarily provided by nurses and healthcare assistants (HCAs) while other healthcare professionals, such as pharmacists, speech and language therapists, dieticians, geriatricians and general practitioners, visited care facilities at varying frequencies [26]. All staff and healthcare personnel providing care on the sites were invited to participate. Implementation and data collection took place from May to November 2019.

### Implementation strategies

A PAR approach was used as an implementation strategy. This enabled researchers and participants to collaborate in the implementation of the guidance, tailored to the needs of site and staff. A situational analysis informed by the CFIR framework [19] and revised/integrated version of framework *Promoting Action on Research Implementation in Health Services* i-PARIHS [24] was conducted pre-implementation at each study site to gain an understanding of the context. The i-PARIHS provides a framework for operationalising action research, identifying facilitation as the active element which integrates the innovation, recipients and context for implementation [24]. The situational analysis encompassed a site profile questionnaire, staff surveys exploring learning needs and the implementation climate, and an audit of practice relating to the guidance implemented at each site (reported in Timmons et al. [26]). The PAR approach involved the setup of work-based learning groups (WBLGs) in which structured discussions took place on how practice could change within the care context to implement the guidance. WBLGs were facilitated by two members of the research team, one of whom was an experienced PAR facilitator. Both were external to the site, with nursing backgrounds and known as ‘external change agents’ within the CFIR framework. A series of five WBLGs were conducted at each site. The first session entailed getting to know the guidance and presentation of site-specific results from the pre-implementation situational analysis. The second and third sessions were focused on how to promote the use of the guidance and development of action plans to progress. The fourth session engaged staff in making the guidance a reality and the final session included reflection and evaluation. The dissemination of learning in WBLGs was encouraged as an additional feature of this approach, where staff were encouraged to communicate their learning to colleagues unable to attend. Champions with a leadership role were also identified at each site to support engagement with the implementation. Champions were Directors of Nursing or Practice Development staff that facilitated the introduction of the guidance at the local level.

### Data collection

Pre-implementation data were collected using staff surveys and audits of practice to inform the situational analysis. Post-implementation, these were repeated, along with interviews with champions to explore their perception of the process of implementation and its effectiveness (Table 1).

**Table 1:** Qualitative and quantitative data collection

	***N*** (pre)	***N*** (post)	Question topics/data
**Staff questionnaire**	69	45	*Pre-implementation* • Age, gender, educational and professional qualifications, number of years working at the site, previous training • Top 3 learning needs in dementia palliative care and top 3 learning needs relating to site guidance topic • VOCALISE tool (measures readiness to implement change) *Post-implementation* • Three learning needs relating to site guidance topic • VOCALISE tool • 23-item tool rating experience of implementation (researcher-developed based on guidance) • Top 3 barriers and top 3 facilitators of implementation
**Audit of practice**	15	15	Audit tool tailored to capture care provided for each guidance topic: hydration and nutrition, pain management or medication management
**Champion interviews**	–	4	• Impression of the project and how it worked • Perspective on the process of WBLGs as an implementation strategy • Use of guidance at site during/after • Future sustainability of practice change and/or reach/embedment of guidance into practice

#### Staff survey

Staff were surveyed pre- and post-intervention. A total of 69 (57% response rate) of staff across the three sites completed the pre-implementation staff survey while 45 (41% response rate) completed the survey post-implementation. Although the survey was distributed to all healthcare professionals at each site, respondents were mainly nurses and HCAs (59 (86%) and 42 (93%) pre-implementation and post-implementation, respectively). The staff demographics are included in Supplementary File 1. The survey packs with a letter of invitation and an information sheet were delivered to each study site by researchers and then distributed to staff by the site champion. Staff were invited to return the surveys within 2 weeks using a secured ballot box located at a designated area accessible to all staff. One reminder was sent before the ballot boxes were removed for data processing and analysis.

The survey included demographic questions (Table 1) and a learning needs questionnaire in which staff were asked to identify their top three learning needs relating to dementia palliative care in general, and relating to the guidance for implementation at their site. The survey also included the Views On Change and Limits in In-patient Settings *(VOCALISE*) self-report measure [30] to assess staff perceived readiness to implement change within their ward. VOCALISE is an 18-item self-report tool, containing three sub-domains: *Powerlessness*, *Confidence* and *De-motivation*, with items rated on a 6-point Likert scale ranging from strongly agree to strongly disagree. It has reasonable test-retest concordance (rho = 0.76) and good internal consistency scores based on factor analysis, with an overall Cronbach’s alpha score of 0.71 reported [26, 30].

In addition to the above, the post-implementation survey included 23 researcher-developed items inviting staff to rate their experience of the implementation strategy and intervention on a 6-point Likert scale from strongly agree to strongly disagree (see items in Supplementary File 2). Staff were also asked to identify the top three barriers and facilitators to implementing the guidance in their setting. A space was also provided for comments.

#### Audit of practice

An audit of resident care charts (*n* = 5) per site equating to 22% of the total number of residents (*n* = 67) was conducted by one researcher prior to implementation and repeated at 1 month following completion of the WBLGs. The five charts were selected by the site champion each time to ensure that they were representative of the range of residents with dementia (mild to severe with varying degrees of complexity). The audit was conducted using a guidance relevant chart review form to capture clinical information on indicative evidence associated with the principles outlined in each guidance document. For example, within the pain chart review form, data were captured on documentation of pain treatment plans linked to the good practice principle of ‘each *patient will have their pain controlled to a degree that is acceptable to them*’.

#### Champion interviews

Post-implementation, champions (*n* = 4), at least one from each site, were interviewed by telephone. Three of the champions were the directors of nursing at each of the study sites and one champion was a practice development co-ordinator based at one of the sites. After gaining consent, the interviews were recorded and transcribed. The objective of these interviews was to explore the champions’ perspectives of the project and implementation process. Question topics are included in Table 1.

### Data analysis

#### Quantitative data

**Fig. 1:**
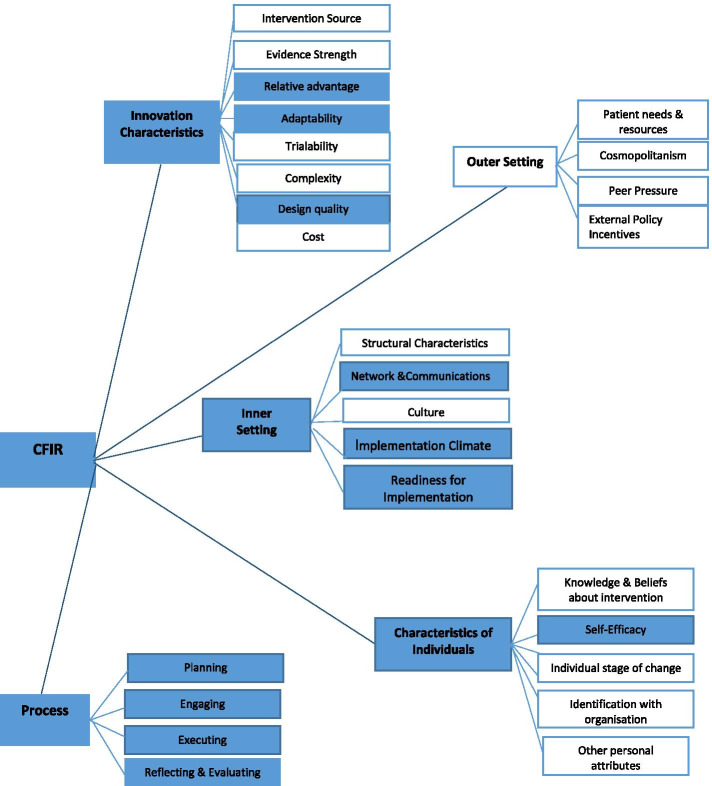
Factors influencing implementation in this study based on the Consolidated Framework for Implementation (CFIR) [18]. Components highlighted in blue were identified as influential in this study while components in white were not readily identified

Comparisons between pre- and post-VOCALISE scores were made using Mann-Whitney *U* tests given that data did not meet parametric assumptions. Scores pre-implementation were compared with all survey responses post-implementation and were also compared with scores from those respondents who attended WBLGs. Comparisons of staff learning needs pre- and post-implementation were made using a chi-square test. Responses from the researcher-developed tool to assess perceptions of implementation were collapsed from the 6-point Likert scale (strongly agree to strongly disagree) into two categories (yes/no) for ease of cohort analysis and frequencies were reported for two groups based on attendance at WBLGs. The facilitators and barriers identified by participants were grouped, checked by a second author and collated. Audit data were narratively described; statistical comparisons were not possible given the small samples (*n* = 5). Data were analysed using SPSS (version 25) and findings mapped to the components of the CFIR (Fig. 1) and are reported narratively according to the CFIR domains.

#### Qualitative data

The anonymised champion interview transcripts and staff survey comments were analysed using deductive content analysis [31]. The components of the CFIR were used as a coding template. Data analysis involved familiarisation with the data, identifying meaning units in the transcripts and notes, re-checking data to ensure that all content relating to the aim had been covered and deciding on the inclusion/exclusion of unmarked text [31]. The initial coding was conducted by SOC, and a sample of transcripts were independently coded by a second author (AC). To foster reflexivity and enhance the consistency of the application of the coding framework [32], coding was compared and the two authors discussed discrepancies to inform the final coding. NVivo 12 was used to support data analysis. The qualitative and quantitative data were assembled under the CFIR components to develop the narrative report according to the CFIR domains.

## Results

The findings include (1) a pre-post comparison of staff learning needs, staff readiness to change and audit of practice and (2) evaluation of the process of implementation through staff survey items and interviews with site champions. Of the 42 nurses and HCAs who completed the post-implementation survey, 31 (74%) attended at least one WBLG session. Findings are presented according to the CFIR framework. Within the CFIR, characteristics of the innovation, the inner setting and the process were most frequently illuminated by the data while the outer setting and characteristics of the individuals received limited attention in the data (influential components of CFIR are highlighted in Fig. 1). This was likely due to the focus of our intervention being site specific and the staff collective rather than individual.

### Innovation characteristics

#### Relative advantage

Participants identified an advantage to implementing the intervention compared to their previous practices. In the post-implementation survey, 100% of staff who attended WBLGs indicated that they had high expectations about both the education and implementation of the guidance in dementia care on their ward; however, this view was less common (56%) amongst those who did not attend WBLGs (tabulation of responses included in Supplementary File 2). Champions reported that the intervention raised awareness of different assessment and management techniques available in each area; it made staff reflect on care and think outside the box and was valued for its orientation towards person-centred care. Specific tools to aid pain assessment were valued such as the Doloplus 2 Scale for patients with cognitive impairment or communication issues. Champions also highlighted that the guidance provided affirmation for the practices they were already engaging in. One champion identified how the guidance was a good back-up to support their approach to care:

Some of us have done dementia and palliative care training and had a background in it already so I suppose what it did was to give us the confidence to keep on doing what we were doing and another way of looking at things. (Champion C, Site 3)

#### Adaptability

In one site, a champion noted that when the guidance was introduced gradually and observed by staff in practice with one resident initially, this provided support for the guidance and encouraged staff to incorporate it.

It had gone from focusing on one residents needs to involving the whole process of review of all residents’ medications. I came away saying, okay, this is something we can start to bring to the attention of other ward managers in the area. (Champion A, Site 1).

#### Design quality and packaging

This intervention included the provision of hard copy guidance documents with integrated fact sheets and assessment tools. Data from post-implementation questionnaires indicated that all staff who attended WBLGs felt that the guidance documents were useful. Seventy per cent (7/10) of those who did not have the opportunity to attend a WBLG also felt the documents were useful, demonstrating reach beyond the WBLGs. Champions mentioned how beneficial it was to have all pertinent information in one document and that this information along with the fact sheets was accessible. However, they noted that the fact sheets had the potential to be lost within the overall guidance document without drawing attention to them as part of the WBLGs. The WBLGs were valued as a way of highlighting critical aspects of the guidance.

It is great to have information in a book and people know where to get it, but the fact sheets can get lost in the pack. This process (WBLGs) brings them to the fore. (Champion A, Site 1)

### Inner setting

#### Networks and communications

Site profiles indicated that allied health professionals from different disciplines visited care settings with varying frequency. A lack of support from other allied health professionals was reported as a barrier to implementation by 26% (*n* = 6) of staff (Table 2). For example, support for medication review requires input of doctors and pharmacists. To a lesser extent, champions also highlighted the influence of support from other allied professionals but did not frame this as a barrier.

**Table 2:** Top barriers and facilitators to implementation identified by nurses/HCAs, number of times barrier/facilitator was identified and number of participants who identified each barrier/facilitator

Barriers	No. times identified	*n*	Facilitators	No. times identified	*n*
Staffing shortage	9	8	Ward manager/clinical nurse manager	12	11
Limited time	7	7	Support of other staff/colleagues	14	9
Support of other allied health professionals^a^	15	6	Team work	3	3
Limited continuity of staff/care	4	4	Leadership	3	2
Engaging resident/challenging behaviour	4	4	Nursing administrative support	2	2
Lack of knowledge	4	2			

*Note*: 23 nurses/HCAs identified at least one barrier and 16 identified at least one facilitator of implementation with a total or 50 barriers and 40 facilitators identified

^a^Allied health professionals identified included doctors, occupational therapists, speech and language therapists, pharmacists and physiotherapists

#### Implementation climate

The implementation climate captures shared openness to change and is reflected in both the qualitative and quantitative data. The qualitative data suggested that champions welcomed education in dementia palliative care: “Education is always important to keep up to date” (Champion B, Site 2). The support and engagement of other colleagues in the process was a prevalent facilitator of implementation identified in staff surveys (*n* = 9) while teamwork was also identified as a facilitator (*n* = 3) (Table 2).

The VOCALISE scale [30] and subscales also shed light on factors affecting implementation within the inner setting (Table 3). Pre and post, participants had negative results for the de-motivation subscale. This subscale refers to the lack of motivation experienced by staff when colleagues are not engaging with the intervention. Overall, there were no statistically significant differences from pre-implementation to post-implementation. However, results indicated reduced de-motivation in those who attended the WBLG in post-implementation data (*Mdn* = 22.00) compared with the pre-implementation (*Mdn* = 19.50), *U* = 497.00, *p* = 0.07, suggesting that engagement in the implementation strategy through a PAR process may have positively impacted on de-motivation.

**Table 3:** Comparison of VOCALISE subscales Pre and Post-implementation

	Pre-implementation			Post-implementation							
	All nurse and HCA (***n*** = 51)			All nurse and HCA (***n*** = 35)				Nurse and HCA who attended WBLGs (***n*** = 26)^a^			
VOCALISE	**Mean (*SD*)**	**Median**	**Interpretive range**	**Mean (*SD*)**	**Median**	**Interpretive range**	***P***	**Mean (*SD*)**	**Median**	**Interpretive range**	***P***
Total	61.25 (9.65)	62	Ambivalent (55–71)	59.66 (10.44)	56	Ambivalent (55–71)	0.50	58.12 (10.82)	55	Ambivalent (55–71)	0.19
Powerlessness	22.61 (4.95)	24	Ambivalent (22–27)	23.34 (5.39)	23	Ambivalent (22–27)	0.94	22.96 (5.92)	22	Ambivalent (22–27)	0.74
De-motivation	21.53 (3.17)	22	Negative (20–30)	20.34 (5.08)	20	Negative (20–30)	0.23	19.73 (5.35)	19.50	Negative (20–30)	0.07
Confidence	17.12 (4.03)	17	Positive (6–18)	15.97 (3.15)	16	Positive (6–18)	0.26	15.42 (2.69)	16	Positive (6–18)	0.14

^a^Nurse and HCA who attended WBLGs (*n* = 26) compared to all nurse and HCA pre-implementation (*n* = 51)

#### Readiness for implementation

Leadership and support from ward managers/CNMs were amongst the most common facilitators of implementation identified by staff. Following implementation, almost all those who attended the WBLGs felt that attendance was supported by management (100%) and agreed that the implementation of guidance received managerial support (97%). For staff that did not attend WBLGs, the percentage expressing the same views was lower 56% (5/9) and 60% (6/10), respectively. This suggests that a small number of staff perceived that there was a lack of management support for implementation which may have influenced their participation.

The availability of resources also influenced the implementation strategy and intervention. In terms of access to knowledge and information, when surveyed, 97% of staff who attended at least one WBLG agreed that enough education sessions were provided. Unsurprisingly, only 40% (4/10) of those who were unable to attend WBLGs agreed with that statement. A small number of staff (*n* = 2) surveyed post-implementation reported lack of knowledge as a barrier to guidance implementation and qualitative data indicated that time to attend the WBLGs was a challenge. Champions discussed the challenge and stress for staff of being needed ‘on the floor’ when residents required high-level support.

Not all staff were able to attend and those that did, felt the stress of catching up on our work once back on the ward (nurse, survey comment).

From a champion perspective, inconsistencies in attendance across WBLG sessions also created difficulty with continuity of learning. In the post-implementation survey, changing staff and lack of continuity of care were reported as barriers to implementation (Table 2).

It worked well I have to say but every time there were different staff going up to the information sessions so that would be an issue. (Champion B, Site 2)

In addition, the powerlessness subscale of the VOCALISE instrument includes factors that influence staff readiness for change including inadequate staffing, time available for implementing changes, and ease of making changes. Our results show participants as ambivalent in relation to powerlessness pre- and post-implementation (Table 3) suggesting conflicted views on their power to implement change. Lack of staff and time were identified as barriers to implementation by 34% (*n* = 8) and 30% (*n* = 7) of participants, respectively.

While it was noted that limited staff availability affected the continuity of the WBLGs, it is not required that the same staff attend all the sessions in the WBLG facilitation process. Information and learning can be disseminated amongst staff via other communication opportunities such as handover. In support of dissemination, 100% of staff who attended WBLGs reported that they had shared the knowledge gained with other colleagues. Additionally, 73% (8/11) of those who did not attend WBLGs had accessed the guidance and the same number expressed confidence in using the guidance. Overall, the number of HCAs and nurses reporting any learning needs relating to the guidance documents decreased (*x*^*2*^ = 6.28, *p* < 0.05) from pre-implementation (*n* = 36, 61%) to post-implementation (*n* = 12, 34%).

### Individual characteristics

Aside from the individual characteristic of self-efficacy, the data does not illuminate individual characteristics as influencing implementation. Both pre- and post-implementation, participants scored positively on the confidence subscale which may contribute to self-efficacy to implement the intervention (see VOCALISE results in Table 3).

### Process

#### Planning

There were some suggestions from both champion interviews and staff surveys that intervention planning could have been improved to take cognisance of issues with staff resources and attendance at WBLGs. Some reported a lack of awareness of WBLG sessions, challenges in freeing staff to attend the groups and challenges for staff on night duty to attend daytime sessions. Champions suggested enhanced communication, to ensure that all staff are made aware of the existence and purpose of WBLG s and a greater involvement of nursing directors/high-level management to help address staff cover and attendance.

#### Engaging

The champions’ account of implementation suggested fidelity to the intended implementation strategy through WBLGs. The external change agents, that is the WBLG facilitators, were positively evaluated. All staff who attended the WBLGs reported satisfaction with the facilitators’ delivery of the education. Staff perceived the facilitators as being enthusiastic and experienced in their fields. Moreover, staff and champions valued how the facilitators engaged them in creative reflection and active thinking about the practical implementation of the guidance in the context of their ward.

I thought it was very interesting. It was great the way that it was done. I thought that the two facilitators on the day for me were good and all the staff would have felt the same. They engaged us well and they got us thinking and I liked the process – I enjoyed it actually. (Champion D, Site 3)

There was an eagerness for facilitators to return to provide further support. The follow-up process was perceived as useful.

From the presentation point of view, everything was made very clear and followed up with action plans. We used these and they followed up with us to see how we got on. (Champion B, Site 2)

The participatory process was valued as it encouraged the engagement of all staff and supported each individual’s input. As described by Champion A (Site 1), diversity in attendance “opened up huge areas and actually the awareness too that other staff have insights as well”. However, other health professionals such as GPs were less engaged and this was suggested as a useful target for future projects. The use of images and ‘evoke’ cards with the groups were valued as a means of encouraging full engagement:

With the cards everybody had to become involved and everybody had to engage. It was good for encouraging feedback really and there was a couple of varied responses that might not have happened without that creativity. (Champion C, Site 3)

#### Executing

In interviews with champions, they all indicated that the guidance was implemented in their sites. There were some differences in the manner in which the guidance was executed. This often took the process of initial trialling of an assessment tool with one or more residents for example and then expansion to others. However, there was also an acknowledgement that not all staff had adopted new techniques as part of the practice. The project was seen as a good starting point, but there was a journey ahead in terms of adopting the guidance consistently in practice. In the case of guidance on hydration and nutrition, staff were reported to be already engaging in most of the guidance recommendations. Nevertheless, the intervention provided affirmation of practice and enhanced awareness.

Data from an audit of the records at each site indicated increased compliance with processes recommended by the guidance both pre- and post-implementation (Table 4). Some items not frequently documented pre-implementation were documented to a greater extent post-implementation, such as discussions with family and residents concerning medications.

**Table 4:** Audit of residents records pre-implementation (*n* = 15) and post-implementation (*n* = 15). Different resident charts were audited pre and post, and thus, direct comparisons were not possible

	Hydration and nutrition	Medication management	Pain assessment and management
Documentation	● In both phases, there was full compliance for assessments of nutritional status. ● There was close to full compliance for recording of weight, safety for eating alone/swallowing status, risk of ulcers/pressure sores and record of dental/oral assessment. ● Discussions/feedback from the resident or their family (dependant on resident’s dementia stage and ability to indicate preferences) were documented in both phases.	● All residents whose charts were reviewed received a medication review in the previous year. ● From pre to post, there were more reviews documented with decision-making about continuation/discontinuation of medication. ● More discussions about medications with resident’s family and with residents who are capable of having a discussion were documented. ● More reviews of antipsychotic prescriptions documented post-implementation. ● More medication reviews of safety to receive medications and covert drug administration documented post-implementation.	● Evidence of adoption of an alternative pain assessment tool for one resident was observed in the post-implementation analysis. Documented evidence of daily frequency of assessment was also noted post-implementation in four of the charts. ● General details about the pain assessment were recorded for all participants, which was the same as the observed pre-implementation audit. ● For site, type and location assessments, there was no change, and for one, i.e. the number of pains, there was an increase in documentation. ● The HCP approach to the pain and treatment plan is the same as pre-implementation with all residents having undergone these assessments. ● Medications and side effects, ongoing assessments and effectiveness of treatment, overall, since the implementation of EBG, there is less evidence of documentation of ongoing assessments and actions to remedy side effects. There was an increase for prescription for breakthrough pain. ● As with the pre-implementation audit, all but one of the residents were unable to be involved in their pain treatment. For the one resident post-implementation audit that could interact with the MDT team, there was no record of any discussions with them about their treatment.

#### Reflecting and evaluating

Champions noted that the intervention had provided an opportunity for direct reflection on practices through the WBLGs. However, the importance of providing feedback from the study findings to staff was stressed to encourage further engagement with the guidance.

I’d like to be able to relay that feedback to the staff and make it available to them. Just to look at your comments and to have a ‘two-way’ connection between the research results to see the staff reaction. (Champion C, Site 3)

## Discussion

This study explored and identified the factors affecting the implementation of evidence-based guidance on dementia palliative care in three long-term care facilities for older people. The implementation of evidence-based guidance was guided by CFIR in conjunction with a participatory work-based learning approach. This implementation approach resulted in reduced staff self-reported learning needs and increased awareness of the guidance for medication review, pain management and hydration and nutrition. However, there was some diversity between the sites dependant on their previous practices. In one site, implementation was facilitated by the introduction of new assessment tools and gradually expanding in their application. This was identified as the beginning of a journey toward improved practices. However, in another site, staff were already using many of the recommended approaches, so our intervention provided affirmation for their practices. While the small-scale audit was primarily used to provide context and inform the implementation strategy, comparisons between the baseline and follow-up audit suggest that the guidance was used but not in all cases. One barrier to implementation related to existing documentation, which did not allow for certain care practices to be documented in a standardised way. For example, there was no designated area in existing documentation for recording assessment of side effects of pain medication, or of total pain assessment. Therefore, even when this was discussed in the WBLGs, the staff were unable to document this practice and change was not captured. This has implications for future nursing documentation and its alignment to evidence-base guidance, actual care provided and sustained practice change. Furthermore, pre-implementation, pain assessment and management was the most frequently identified staff learning need [26]. This was similarly identified as a prominent learning need in a study in the Netherlands [33] suggesting that pain assessment and management may be a particularly challenging aspect of dementia palliative care that requires longer-term implementation strategies.

Our findings suggest that the PAR approach using WBLGs served as a positive influence on implementation bringing guidance into action through tailoring the intervention to the site-specific context and level of progress. As identified in previous research, the enthusiasm and experience of facilitators contributed positively to the participatory process [20] and thus facilitated implementation. Similarly, staff in this study felt that the process of implementation encouraged them to think about how they could practically implement the guidance which corresponds with reported positive impacts of experiential learning approaches in dementia education [20]. Staff identified many positive impacts of using the evidence-based guidance including anticipated improvement in resident outcomes and in person-centred care, which reiterates the importance of considering the relevance of the learning material to the participants [20] and of stakeholders’ perception of the relative advantage of implementing the guidance [19]. Findings highlighted the WBLGs as key facilitators of implementation and use of the guidance, by drawing attention to important parts of the guidance, such as the tool kits and fact sheets as well as setting action plans/goals to implement changes.

In addition, our findings suggest that the visible engagement of other staff enhanced motivation to engage in the intervention. Pre-implementation, negative results for the de-motivation subscale of the VOCALISE instrument indicated that staff may be negatively influenced by the lack of engagement of other staff. This score improvement in the post-implementation phase, suggesting that the implementation process through WBLGs, may have somewhat tackled staff de-motivation. New practices introduced on a small scale and observed to be useful encouraged staff to engage and expand their use. This corresponds with the CFIR construct of trialability which has been shown to have a strong positive association with effective implementation [19]. Our findings also suggest that a combination of modelling change and feedback of positive outcomes encouraged other staff to implement the guidance. This also corresponds with the concept of observability, where being able to directly observe the impact of change, and facilitates the longer-term adoption and retention of evidence-based guidelines in long-term care settings [34]. WBLGs provided the platform for this communication and exchange but this strategy was time limited and it was clear from the data that staff preference was for the continuity of WBLGs and further feedback beyond the duration of the project. This time-limited implementation strategy may be a barrier to sustainability.

Although WBLGs were perceived as positive from an implementation perspective, staff attendance at the WBLGs was also identified as a barrier to implementing the guidance. From the champions and staff perspectives, inconsistent attendance led to challenges with continuity of learning across sessions and attendance sometimes led to stress for staff when trying to catch up on work afterward. Surr et al. [21] previously highlighted that opportunity factors such as lack of time and staff are prevalent barriers to implementing dementia care interventions in nursing homes. Effective implementation is influenced by the degree of compatibility with existing workflow and resources [19]. A study using the VOCALISE instrument in the inpatient mental health context found that staff attitudes to change worsened over the course of an intervention due to a lack of resources to implement the change [35]. In our study, attitudes to implementing changes, as captured by the VOCALISE instrument, were similar across time points. However, resources posed a challenge to attending the WBLGs and implementing the intervention. While it was not required that all staff attend the WBLGs, qualitative data suggests that staff valued the opportunity to participate. This highlights the importance of ensuring equitable access to WBLGs for all staff involved in change. Staff suggested more planning and engagement with higher-level management, to enable staff to attend and have their workload covered. Similar to previous research in long-term care [34], this highlights the important role of organisational factors, such as staffing levels and workload, and organisational change through effective clinical leadership in facilitating the implementation process.

Similarly, support from other health professionals influenced implementation. Staff were positive about diversity within the WBLGs with nurses, HCAs, allied health professionals and ancillary staff. However, targeting healthcare professionals such as GPs and pharmacists would be useful. In some cases, staff felt that support was inadequate from other health professionals and this was a barrier to implementing the guidance which relied on their assistance. These findings are reflected in previous research where staff were frustrated with PAR when it did not involve staff who played an important role in achieving the targeted processes and outcomes [36]. Overall, our results suggest a need for greater organisational investment and involvement of all key stakeholders in the implementation process. Recent work drawing on international literature and national consultation in Ireland identified collaborative practice as the supportive platform upon which evidence-based practice is implemented to enhance clinical effectiveness across professional disciplines [37].

The CFIR [19] provided a useful framework to identify influential factors pre-implementation to guide the implementation, and to examine the process post-implementation. A strength of the study is that CFIR along with i-PARIHS [26] was used from the conception of the research, to inform the intervention and to evaluate the process of implementation, given that many studies only employ CFIR in post hoc analysis [38]. Most of the influential factors in this study concerned the intervention characteristics, the inner setting and the process of implementation, and there was little reference to factors in the outer setting or individual characteristics. This pattern has been noted in other implementation studies in the healthcare context [39]. Individual capability factors are not often identified as barriers compared to opportunity or motivation factors in dementia education literature [20, 21]. Maybe individual characteristics have a more indirect effect on implementation compared to other factors. A study of the application of CFIR to a complex nursing intervention identified that individual characteristics and intervention characteristics exerted influence on the process via the inner setting and particularly the construct ‘tension for change’ [40].

The data collection tools in this study were not directed at culture, individual characteristics or the outer setting which may affect the extent to which these components were identified. Future research should further assess the salience of these constructs in implementing dementia palliative care. While the sample for the audit was small, it was notable that some current care documentation could not be altered to reflect the guidance which may have affected the ability to identify practices relating to guidance, particularly in the case of pain management.

### Limitations

This study pertains to data from three long-term care facilities in Ireland and sample sizes were small so findings may not generalise. Independent sample tests were used to compare staff measures pre- and post-implementation due to the reduced sample size post-implementation, though many staff participated in both. Furthermore, the study primarily relied on staff self-report and to a lesser extent on the audit of resident charts. Studies incorporating observation or evaluation from other stakeholders’ perspectives such as carers, other health professionals or higher-level management may provide a more complete picture of the implementation process and outcomes. Economic evaluation was not possible but should be considered in the future guidance implementation.

## Conclusion

The CFIR provided a useful framework for analysing the implementation context and informed the strategy for implementing dementia palliative care guidance. The participatory action research approach involving WBLGs, tailored to the context of each setting, was identified as a key factor that facilitated the implementation of the guidance. Post-implementation, CFIR helped identify factors influencing implementation including features of the guidance, the inner setting of the long-term care facility and the process of engaging with stakeholders. Recommendations for practice include greater organisational investment and involvement of all key stakeholders in the implementation process. Valued components of the PAR process were the engagement of staff of different backgrounds who have a role in change, a practical focus with action plans, and opportunities to observe the impact of the guidance and share this with other staff. This suggests that putting a mechanism in place to continue the WBLGs may help to sustain the shift to reflection and the use of evidence-based guidance in practice. However, it is important to ensure equitable access to WBLGs for all staff involved in change. This will involve early engagement with higher-level management, to enable staff to attend.

Factors identified as potential barriers to implementation of guidance for dementia palliative care were limited staff time and support for the implementation from other healthcare professionals. This emphasises the importance of organisational factors and managerial support in facilitating the implementation process. In the future implementation of guidance in palliative care, we recommend the use of the CFIR to identify context-specific factors affecting the implementation that can be targeted in a participatory approach.

## Supplementary Information

**Additional file 1: Table S2.** Demographics of staff completing questionnaires pre and post implementation.

**Additional file 2..** Comparison of Staff Perception of the Implementation of EBG: Comparison Between Attenders and Non-Attenders at the EBG Education Sessions. Proportion of staff who strongly agree/agree/slightly agree with the following statements.

## Data Availability

The datasets generated and/or analysed during the current study are not publicly available due to the confidential nature of the data but are available from the corresponding author on reasonable request.
