# Supplementary material for: Implementation of evidence-based guidance for dementia palliative care using participatory action research: examining implementation through the Consolidated Framework for Implementation Research (CFIR)
Source: Implement Sci Commun. 2021 Dec 11;2:137. doi: 10.1186/s43058-021-00241-7 (PMC8665505; doi:10.1186/s43058-021-00241-7)
Supplement: Supplementary file 1 — Additional file 1: Table S2. Demographics of staff completing questionnaires pre and post implementation. [file 43058_2021_241_MOESM1_ESM.docx]

**Table 2.** Demographics of staff completing questionnaires pre and post implementation.

| **Demographics** | **Total Pre-Implementation**  **N=69** | **Post Implementation**  **N=45** |
| --- | --- | --- |
| **Age** |  |  |
| <30 yrs | 10 (14%) | 2 (4%) |
| 30-39 yrs | 20 (29%) | 14 (31%) |
| 40-49 yrs | 17 (25%) | 14 (31%) |
| ≥ 50 yrs | 22 (32%) | 15 (33%) |
| **Gender** |  |  |
| Male | 8 (12%) | 8 (18%) |
| **Highest Educational Level** |  |  |
| Professional Qualification | 32 (47%) | 23 (51%) |
| Bachelor degree | 15 (22%) | 10 (22%) |
| Postgraduate Qualification | 21 (31%) | 12 (27%) |
| **Category of Staff** |  |  |
| HCA | 27 (39%) | 20 (44%) |
| Nurse | 32 (46%) | 22 (49%) |
| Other HCP | 10 (14%) | 3 (7%) |
| **No. of Years Working at Site** |  |  |
| <5 yrs | 36 (53%) | 15 (33%) |
| 5-14 yrs | 15 (22%) | 12 (27%) |
| ≥15 yrs | 17 (25%) | 18 (40%) |
| **Site*** |  |  |
| Site 1 | 32 (46%) | 15 (33%) |
| Site 2 | 16 (23%) | 17 (38%) |
| Site 3 | 17 (25%) | 13 (29%) |
|  |  |  |

*Four questionnaires (6%) could not be associated with a site.
