# Supplementary material for: Implementation of evidence-based guidance for dementia palliative care using participatory action research: examining implementation through the Consolidated Framework for Implementation Research (CFIR)
Source: Implement Sci Commun. 2021 Dec 11;2:137. doi: 10.1186/s43058-021-00241-7 (PMC8665505; doi:10.1186/s43058-021-00241-7)
Supplement: Supplementary file 2 — Additional file 2.. Comparison of Staff Perception of the Implementation of EBG: Comparison Between Attenders and Non-Attenders at the EBG Education Sessions. Proportion of staff who strongly agree/agree/slightly agree with the following statements. [file 43058_2021_241_MOESM2_ESM.docx]

Comparison of Staff Perception of the Implementation of EBG: Comparison Between Attenders and Non-Attenders the EBG Education Sessions with Non-Attenders. Proportion of staff who strongly agree/agree/slightly agree with the following statements.

| Statement | Did not Attend (N=11) | | Attended  (N=31) | |
| --- | --- | --- | --- | --- |
|  | **N** | **%** | **N** | **%** |
| I heard from a colleague about the WBLGs (N=41) | 10 | 91% | 27 | 90% |
| I am aware of the guidance documents (N=42) | 9 | 82% | 31 | 100% |
| I have gained knowledge in EBP since the implementation (N=42) | 7 | 64% | 31 | 100% |
| I have shared knowledge with other colleagues (N=42) | 6 | 55% | 31 | 100% |
| I have accessed the guidance documents onsite to inform my practice (N=41) | 8 | 73% | 30 | 100% |
| I'm confident using guidance documents to inform practice (N=41) | 8 | 73% | 30 | 100% |
| I participated in at least one of the activities (N=39) | 3 | 30% | 28 | 97% |
| I am more aware of the EBG Implemented onsite (N=40) | 5 | 50% | 30 | 100% |
| The information I received is relevant (N=41) | 6 | 60% | 31 | 100% |
| The activities & examples were appropriate (N=40) | 3 | 38% | 31 | 100% |
| The guidance documents and WBLG report are useful (N=42) | 7 | 70% | 31 | 100% |
| High expectations about the Education & Implementation (N=39) | 5 | 56% | 30 | 100% |
| Satisfaction with Education/Implementation of EBG (N=41) | N/A |  | 31 | 100% |
| Satisfaction with the facilitators delivery of the education (N=38) | N/A |  | 31 | 100% |
| There were enough education sessions provided (N=40) | 4 | 40% | 29 | 97% |
| Attendance was supported by management (N=40) | 5 | 56% | 31 | 100% |
| I have become more innovative in my practice (N=41) | 7 | 70% | 30 | 97% |
| I have enough support to apply EBG in my own practice (N=41) | 6 | 60% | 31 | 100% |
| Management have supported the implementation N=40) | 6 | 60% | 29 | 97% |
| There was enough time to implement the EBG (N=41) | 5 | 50% | 29 | 94% |
| Patient care on the ward has improved because of this implementation (N=40) | 5 | 56% | 31 | 100% |
| We work more closely as a team to implement the best possible care to the residents (N=41) | 7 | 70% | 31 | 100% |

Abbreviations: EBG=Evidence based guidance; WBLG=Work based learning group
